# Supplementary material for: Evolution of gut microbiota across honeybee species revealed by comparative metagenomics
Source: Nat Commun. 2025 Oct 13;16:9069. doi: 10.1038/s41467-025-64115-5 (PMC12518797; doi:10.1038/s41467-025-64115-5)
Supplement: Supplementary file 2 — Description of Additional Supplementary Files [file 41467_2025_64115_MOESM2_ESM.pdf]

Description of Additional Supplementary Files

Supplementary Data 1.
Sample collection information and metadata.

Supplementary Data 2.
Metadata about MAGs including fields (in bold) required by the MIMAG standards.

Supplementary Data 3.
Sequencing depth and Host and MAG database mapping information.

Supplementary Data 4.
Result of multivariate analyses (PERMANOVA test) applied on strain-level profiles for each bacterial species. Column n displays the number of samples in which the species was detected with enough coverage to test for the effect of host species or country on strain-level in a PERMANOVA analysis and the p-value and omega squared values for the respective tests are shown. Host specificity is based on the Rohde's index if strain-level comparison is not available. Values < 1 for which there were not enough samples with good enough coverage for strain-level comparison are considered generalist if there is enough coverage, they are labelled specialist if detected in only one host species, and shared (strain-level specialist) if found in more than one host species but the PERMANOVA test is significant for effect of host species.

Supplementary Data 5.
All nodes in original trees identified by hommola test as significant ($p < 0.05$) with the line separating those that are detected with strict thresholds (above the line). sym_subtree displays a Newick string of the subtree at the node being considered. host_tip span is the number of host species represented in the subtree, sym_tips_count the number of MAGs in the subtree.

Supplementary Data 6.
Number of nodes tested and found significant under various thresholds. "# significant" is the number of nodes under the respective threshold, "median # significant" is the median number of significant nodes in (N=100) second-order permutation test comparisons with randomly shuffled host tip labels and "SD # significant" is the standard deviation.

Supplementary Data 7.
Tip-tip distance between MAGs of the genus in nucleotide tree constructed using 120 core genes (bac120 as identified in GTDB-tk).
