# Supplementary material for: Evolution of gut microbiota across honeybee species revealed by comparative metagenomics
Source: Nat Commun. 2025 Oct 13;16:9069. doi: 10.1038/s41467-025-64115-5 (PMC12518797; doi:10.1038/s41467-025-64115-5)
Supplement: Supplementary file 10 — Reporting Summary [file 41467_2025_64115_MOESM10_ESM.pdf]

nature portfolio

Corresponding author(s): Philipp Engel

Last updated by author(s): 2025/08/21

Reporting Summary

Nature Portfolio wishes to improve the reproducibility of the work that we publish. This form provides structure for consistency and transparency in reporting. For further information on Nature Portfolio policies, see our Editorial Policies and the Editorial Policy Checklist.

Statistics

For all statistical analyses, confirm that the following items are present in the figure legend, table legend, main text, or Methods section.

n/a	Confirmed	
☐	☒	The exact sample size (*n*) for each experimental group/condition, given as a discrete number and unit of measurement
☐	☒	A statement on whether measurements were taken from distinct samples or whether the same sample was measured repeatedly
☐	☒	The statistical test(s) used AND whether they are one- or two-sided<br>*Only common tests should be described solely by name; describe more complex techniques in the Methods section.*
☐	☒	A description of all covariates tested
☐	☒	A description of any assumptions or corrections, such as tests of normality and adjustment for multiple comparisons
☐	☒	A full description of the statistical parameters including central tendency (e.g. means) or other basic estimates (e.g. regression coefficient) AND variation (e.g. standard deviation) or associated estimates of uncertainty (e.g. confidence intervals)
☐	☒	For null hypothesis testing, the test statistic (e.g. *F*, *t*, *r*) with confidence intervals, effect sizes, degrees of freedom and *P* value noted<br>*Give P values as exact values whenever suitable.*
☒	☐	For Bayesian analysis, information on the choice of priors and Markov chain Monte Carlo settings
☒	☐	For hierarchical and complex designs, identification of the appropriate level for tests and full reporting of outcomes
☐	☒	Estimates of effect sizes (e.g. Cohen's *d*, Pearson's *r*), indicating how they were calculated

Our web collection on statistics for biologists contains articles on many of the points above.

Software and code

Policy information about availability of computer code

Data collection

Raw metagenomic data has been deposited to the NCBI Sequence Read Archive (SRA) under the Project ID PRJNA1157353. The collection of MAGs, some important intermediate files, and tables can be found in the Zenodo (https://zenodo.org/doi/10.5281/zenodo.13732977) repository. No software was used in the data collection phase which involved processing of honeybee samples and sequencing.

Data analysis

Code and details of parameters and software tools used are available on GitHub [https://github.com/Aiswarya-prasad/honeybee-cross-species-metagenomics] and archived at the Zenodo repository [https://zenodo.org/doi/10.5281/zenodo.13732977].
Workflow & environment
Snakemake v7.28.1
Conda v23.3.1 (used to build envs)
Mamba v1.4.2 (used to build envs)
Linux vkernel 4.18.0-477.64.1.el8_8.x86_64

Read trimming / QC / initial profiling
Trimmomatic v0.39
BBTools (bbmap, bbnorm, etc.) v39.01
mOTUs v3.1.0

Host/microbe read mapping
BWA-MEM v0.7.17
Bowtie2 v2.5.1

Assembly & binning
metaSPAdes v3.15.3
MetaBAT2 v2.12.1

MAG quality, dereplication & taxonomy
CheckM v1.2.2
dRep v3.4.3
GTDB-Tk v2.3.2

Classification / profiling (reads & contigs)
Kaiju (nr database) v1.9.2
Kraken2 (DB incl. bacteria/viruses added honeybee genome) v2.1.3
InStrain v1.8.0

Phylogenomics & alignments
OrthoFinder v2.5.5
MAFFT v7.520
MACSE v2.07
IQ-TREE v2.2.2.7

Gene prediction, functional annotation & pathway inference
Prodigal v2.6.3
Whokaryote v1.1.2
DRAM v1.5.0
Cayman (CAZyme assignment) v0.9.2
BEDtools v2.31.0
MinPath v1.4

Statistical modeling / multivariable associations
MaAsLin2 v1.16.0

Community ecology & distances (R)
vegan v2.6-4
betapart v1.5.6
MicEco (adonis_OmegaSq function) v0.9.15

Visualisation & trees
iTOL (Interactive Tree of Life, web tool) v6 / now, v7

Programming languages (used for analysis scripts)
R v4.3.1
Python 3.10

For manuscripts utilizing custom algorithms or software that are central to the research but not yet described in published literature, software must be made available to editors and reviewers. We strongly encourage code deposition in a community repository (e.g. GitHub). See the Nature Portfolio guidelines for submitting code & software for further information.

Data

Policy information about availability of data

All manuscripts must include a data availability statement. This statement should provide the following information, where applicable:

- Accession codes, unique identifiers, or web links for publicly available datasets
- A description of any restrictions on data availability
- For clinical datasets or third party data, please ensure that the statement adheres to our policy

Data availability: Raw metagenomic data and metagenome-assembled genomes have been deposited to the NCBI Sequence Read Archive (SRA) under the Project ID PRJNA1157353. The collection of MAGs, some important intermediate files, and tables can be found in the Zenodo (https://zenodo.org/doi/10.5281/zenodo.13732977) repository.

Research involving human participants, their data, or biological material

Policy information about studies with human participants or human data. See also policy information about sex, gender (identity/presentation), and sexual orientation and race, ethnicity and racism.

Reporting on sex and gender	n/a
Reporting on race, ethnicity, or other socially relevant groupings	n/a

Population characteristics	n/a
Recruitment	n/a
Ethics oversight	n/a

Note that full information on the approval of the study protocol must also be provided in the manuscript.

Field-specific reporting

Please select the one below that is the best fit for your research. If you are not sure, read the appropriate sections before making your selection.

☐ Life sciences ☐ Behavioural & social sciences ☒ Ecological, evolutionary & environmental sciences

For a reference copy of the document with all sections, see nature.com/documents/nr-reporting-summary-flat.pdf

Ecological, evolutionary & environmental sciences study design

All studies must disclose on these points even when the disclosure is negative.

Study description	Study of the gut microbiota of honeybee species from South Asia. Individuals were sampled for several colonies of five different species. All individuals of a species were considered as a treatment group for comparisons regardless of their colony affiliation. Only five bees were collected from each colony ensuring that each group contained representatives from several different colonies. All the details of our study design are presented in Figure 1 of the manuscript.
Research sample	Hindguts of individual honeybees from five different species across locations in Malaysia and India. Our sampling strategy was designed to cover several bees of each species of honeybee from their current habitats across Malaysia and in South India. Hindguts were dissected and processed for sequencing as they contain the majority of the bacterial biomass and ensure that host tissue that is not known to harbor gut microbiota (such as malphigian tubules) are removed minimizing host DNA in the final library.
Sampling strategy	Honeybees were collected from forty different colonies of five different bee species at different sites in India and Peninsular Malaysia. Five bees per colony were sampled as collectors curves from previous study (Ellegaard et al) indicated that more than half of the diversity of a colony is represented by five bees. Further we wanted to maximize our coverage across colonies to represent a more robust sampling of the species rather than a deep sampling of one colony as done in previous studies. We also sampled and sequenced individual bees to capture fine strain-level patterns across individuals which are lost if samples are pooled before sequencing. Further details on sampling approach are presented in the methods section of the manuscript.
Data collection	Sampling and collection of Information about the sampling location and colony presented in Supplementary Data 1, was carried out by the researchers sampling in the respective locations (ADP in Malaysia, RS and AS in India) and noted down in pen and paper and then subsequently transferred to electronic medium. DNA was isolated from bees stored in Ethanol and was kept at -80C and sent for Illumina sequencing.
Timing and spatial scale	All samples were collected between August 2021 and October 2022. Each colony was only sampled once for the study.
Data exclusions	One sample was excluded because the host identity of the samples could not be determined due to a mix up during sample preparation. Those samples (n=5) excluded in downstream processing are mentioned in the manuscript.
Reproducibility	Additional samples of each colony are stored in EtOH for re-analysis. DNA of the analyzed samples is stored at -80C. Code of the entire analysis is available and will allow reproduction of the results based on the same samples.
Randomization	Bees were randomly collected from a given colony. Due to morphological differences, it is obvious which species each individual belongs to. For further processing of samples after dissections, such as DNA extractions samples were randomized and DNA was isolated in batches each containing samples from each of the different species to mitigate batch effects.
Blinding	The samples were not blinded during DNA extraction.

Did the study involve field work? ☒ Yes ☐ No

Field work, collection and transport

Field conditions	No observational or other data collected in the field to directly inform the results. Samples were transported and stored until processing immediately after collection. Relevant information about the location of the hive sampled is available in the metadata table listed as Supplementary Data 1.
Location	Detailed information about sampling locations is available in the metadata table listed as Supplementary Table S1.
Access & import/export	In Malaysia, sampling involved the collection of honeybees from reserved forest areas. The permit application was approved by the Perak State Forestry Department to collect the samples from Temenggor forest with the approval number: JH/100 Jld.31(46). Transfer of biological material was carried out under the Access and benefit-sharing (ABS) act, issued by Ministry of Natural Resources and Environmental Sustainability (NRES), Malaysia, April 2019. Reference number: 676216. In India, honeybee sampling

	was conducted from colonies on the campus of the National Centre for Biological Sciences – TIFR or colonies from local beekeepers by Indian researchers. All sampling and processing of biological material was carried out in India, and no material was exported. Publication of this study and data are exempt from prior approval under Section 4 of the Biological Diversity Act, 2002 (as amended in 2023).
Disturbance	A bee colony contains a 1000 to many thousands of worker bees. We sampled about 50 bees per colony which does not represent a major distrubance of the colony. Some colonies of Apis dorsata were collected during the honey sampling of native Orang Asli community in Malaysia. Colonies from urban locations, bees were collected when the colony was being relocated to forested areas by local honeybee conservation group volunteers.

Reporting for specific materials, systems and methods

We require information from authors about some types of materials, experimental systems and methods used in many studies. Here, indicate whether each material, system or method listed is relevant to your study. If you are not sure if a list item applies to your research, read the appropriate section before selecting a response.

Materials & experimental systems

n/a	Involved in the study
☒	☐ Antibodies
☒	☐ Eukaryotic cell lines
☒	☐ Palaeontology and archaeology
☐	☒ Animals and other organisms
☒	☐ Clinical data
☒	☐ Dual use research of concern
☒	☐ Plants

Methods

n/a	Involved in the study
☒	☐ ChIP-seq
☒	☐ Flow cytometry
☒	☐ MRI-based neuroimaging

Animals and other research organisms

Policy information about studies involving animals; ARRIVE guidelines recommended for reporting animal research, and Sex and Gender in Research

Laboratory animals	No laboratory animals were used in the study
Wild animals	Apis dorsata; Apis mellifera, Apis cerana, Apis florea, Apis adreniformis
Reporting on sex	female (sterile worker bees)
Field-collected samples	Honeybees were captured from their hive in an aerated box until they were brought to a laboratory. At the laboratory they were placed on ice until they stopped moving and then stored in ethanol in -20 degrees until they were used for gut dissection.
Ethics oversight	Ethical approval was not required

Note that full information on the approval of the study protocol must also be provided in the manuscript.

Plants

Seed stocks	*Report on the source of all seed stocks or other plant material used. If applicable, state the seed stock centre and catalogue number. If plant specimens were collected from the field, describe the collection location, date and sampling procedures.*
Novel plant genotypes	*Describe the methods by which all novel plant genotypes were produced. This includes those generated by transgenic approaches, gene editing, chemical/radiation-based mutagenesis and hybridization. For transgenic lines, describe the transformation method, the number of independent lines analyzed and the generation upon which experiments were performed. For gene-edited lines, describe the editor used, the endogenous sequence targeted for editing, the targeting guide RNA sequence (if applicable) and how the editor was applied.*
Authentication	*Describe any authentication procedures for each seed stock used or novel genotype generated. Describe any experiments used to assess the effect of a mutation and, where applicable, how potential secondary effects (e.g. second site T-DNA insertions, mosiacism, off-target gene editing) were examined.*
